# Pain-related increase of excitatory transmission and decrease of inhibitory transmission in the central nucleus of the amygdala are mediated by mGluR1

**DOI:** 10.1186/1744-8069-6-93

**Published:** 2010-12-16

**Authors:** Wenjie Ren, Volker Neugebauer

**Affiliations:** 1 Department of Neuroscience & Cell Biology, The University of Texas Medical Branch, Galveston, Texas 77555-1069, USA

## Abstract

Neuroplasticity in the central nucleus of the amygdala (CeA), particularly its latero-capsular division (CeLC), is an important contributor to the emotional-affective aspects of pain. Previous studies showed synaptic plasticity of excitatory transmission to the CeLC in different pain models, but pain-related changes of inhibitory transmission remain to be determined. The CeLC receives convergent excitatory inputs from the parabrachial nucleus in the brainstem and from the basolateral amygdala (BLA). In addition, feedforward inhibition of CeA neurons is driven by glutamatergic projections from the BLA area to a cluster of GABAergic neurons in the intercalated cell masses (ITC). Using patch-clamp in rat brain slices we measured monosynaptic excitatory postsynaptic currents (EPSCs) and polysynaptic inhibitory currents (IPSCs) that were evoked by electrical stimulation in the BLA. In brain slices from arthritic rats, input-output functions of excitatory synaptic transmission were enhanced whereas inhibitory synaptic transmission was decreased compared to control slices from normal untreated rats. A non-NMDA receptor antagonist (NBQX) blocked the EPSCs and reduced the IPSCs, suggesting that non-NMDA receptors mediate excitatory transmission and also contribute to glutamate-driven feed-forward inhibition of CeLC neurons. IPSCs were blocked by a GABAA receptor antagonist (bicuculline). Bicuculline increased EPSCs under normal conditions but not in slices from arthritic rats, which indicates a loss of GABAergic control of excitatory transmission. A metabotropic glutamate receptor subtype 1 (mGluR1) antagonist (LY367385) reversed both the increase of excitatory transmission and the decrease of inhibitory transmission in the arthritis pain model but had no effect on basal synaptic transmission in control slices from normal rats. The inhibitory effect of LY367385 on excitatory transmission was blocked by bicuculline suggesting the involvement of a GABAergic mechanism. An mGluR5 antagonist (MTEP) inhibited both excitatory and inhibitory transmission in slices from normal and from arthritic rats. The analysis of spontaneous and miniature EPSCs and IPSCs showed that mGluR1 acted presynaptically whereas mGluR5 had postsynaptic effects. In conclusion, mGluR1 rather than mGluR5 can account for the pain-related changes of excitatory and inhibitory synaptic transmission in the CeLC through a mechanism that involves inhibition of inhibitory transmission (disinhibition).

## Background

Pain has a strong emotional component and is significantly associated with anxiety and depression. The amygdala plays a key role in emotional learning and memory as well as in affective disorders [1-4] and is also important for the emotional-affective dimension of pain and pain modulation [5-8]. Pharmacologic inhibition of amygdala hyperactivity has been shown to decrease nocifensive and affective responses in animal pain models [5,8-13]. Conversely, pharmacologic activation can produce pain behavior even in the absence of tissue injury [14-17].

The amygdala consists of several anatomically and functionally distinct nuclei [2,18]. The laterocapsular division of the central nucleus (CeLC) has been termed the "nociceptive amygdala" because it receives nociceptive-specific information from the spinal cord and brainstem (external parabrachial area, PB) and the vast majority of CeLC neurons respond exclusively or preferentially to noxious stimuli [5,8,19]. Synaptic plasticity of PB inputs to the CeLC has been shown in models of arthritic pain [20-23], visceral pain [24] and chronic neuropathic pain [25] and is associated with pain-related central sensitization of CeLC neurons [21,26-31]. Highly processed multimodal, including nociceptive, information reaches the CeLC from thalamus and cortex through the lateral-basolateral (LA-BLA) network [5,8]. The LA-BLA circuitry is critical for the emotional evaluation of sensory stimuli and for acquisition and consolidation of aversive associations [2,3,32,33]. Our previous studies showed pain-related synaptic plasticity of excitatory transmission at the LA-BLA and BLA-CeLC synapses [10,20,23]. The BLA can influence CeA processes via direct glutamatergic projections and through indirect disynaptic routes involving GABAergic neurons in the intercalated cell masses (ITC) that project to the CeA [2,32,34]. Activation of inhibitory ITC neurons and subsequent inhibition of CeA neurons has been suggested to play an important role in fear extinction [2,35]. However, the role of synaptic inhibition of CeLC neurons in pain-related plasticity remains to be determined and was addressed in this study.

Another focus of this study was on the involvement of group I metabotropic glutamate receptors (mGluRs) in synaptic inhibition of CeLC neurons, because our previous studies showed that these receptors are important modulators of excitatory synaptic transmission in the CeLC [20]. Group I mGluRs comprise mGluR1 and mGluR5 subtypes and are involved in neuroplasticity associated with normal brain functions as well as in neurological and psychiatric disorders [36-39] and in pain mechanisms [40-42]. Group I mGluRs play a critical role in pain-related central sensitization of amygdala neurons [20,27] and in amygdala-mediated pain behaviors [9,15,43]. Using patch-clamp recordings in brain slices from arthritic rats (kaolin-carrageenan model) and from controls, we measured and compared pain-related changes in inhibitory and excitatory transmission from the BLA to the CeLC and the contribution of group I mGluRs to these changes.

## Results

### Pain-related increase of excitatory transmission and decrease of glutamate-driven inhibitory transmission in CeLC neurons

**Figure 1:**
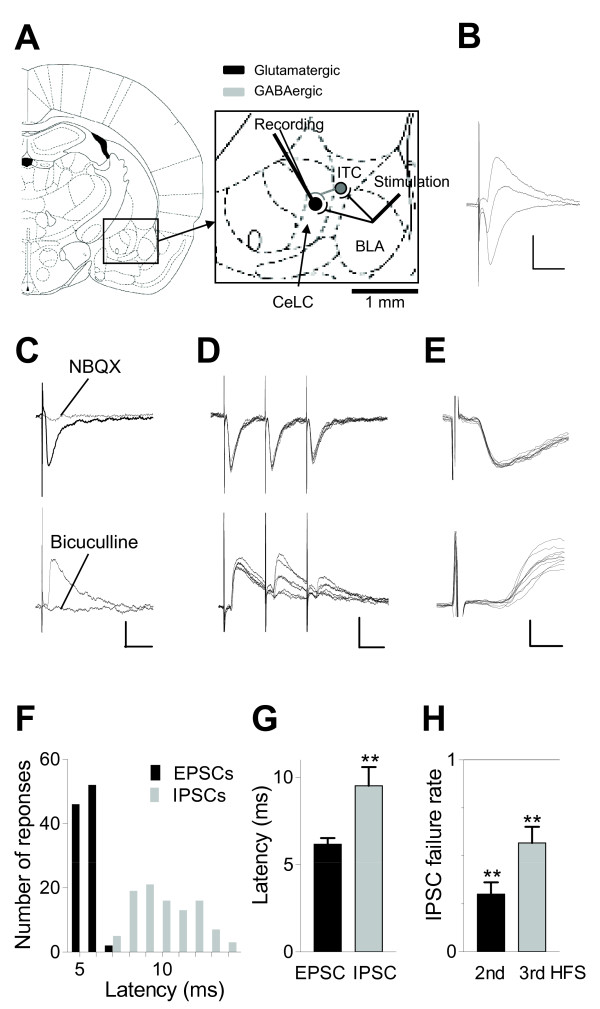
**Monosynaptic excitatory and polysynaptic inhibitory synaptic transmission in CeLC neurons**. (**A**) Coronal brain slices containing the right amygdala were obtained from normal rats and arthritic rats 4-6 h after injections of kaolin and carrageenan (K/C) into the left knee joint. Magnified area shows position of the patch-clamp electrode ("Recording") in the CeLC; stimulation electrode in the BLA to activate direct glutamatergic projections and indirect disynaptic connections that involve GABAergic neurons in the intercalated cell masses (ITC). Diagrams are from [66]. (**B**) Biphasic synaptic responses were evoked at different holding potentials (-70, -30, and 0 mV). (**C**) Individual traces (average of 8-10) of synaptic responses evoked at -70 mV (downward deflections, EPSCs; inhibited by NBQX, 10 μM) and at 0 mV (upward deflections, IPSCs; blocked by bicuculline, 10 μM). Scale bars, 50 pA, 30 ms. (**D**) Monosynaptic EPSCs, but not polysynaptic IPSCs, follow high-frequency stimulation (20 Hz; 6 individual traces each). Scale bars, 50 pA, 30 ms. (**E**) Individual EPSCs and IPSCs evoked with twice-threshold stimulation (30 sweeps each). Latencies of IPSCs were longer and more variable. Calibration: 50 pA, 3 ms. (**F**) Distribution of EPSC and IPSC latencies measured from stimulus artifact to onset of synaptic current in one neuron (n = 100 events). (**G**) Bar histograms show average latencies (means ± SE) of EPSCs and IPSCs in 10 neurons. ** P < 0.01, paired t-test. (**H**) Bar histograms show the number of neurons that did not respond to the second or third high-frequency stimulus (HFS, 20 Hz). The failure rate is normalized for each neuron and averaged across the sample of neurons (n = 15; 0, no failure; 1, no IPSC). ** P < 0.01, compared to 1^st ^stimulus (no failure), Dunnett's multiple comparison test.

Excitatory and inhibitory postsynaptic currents (EPSCs and IPSCs, respectively) were evoked in CeLC neurons by electrical stimulation in the BLA (Figure 1A and 1B). Monosynaptic EPSCs recorded in voltage-clamp at -70 mV were mediated by non-NMDA receptors because they were completely blocked by NBQX (10 μM, Figure 1C). IPSCs recorded at 0 mV holding potential were blocked by a GABAA receptor antagonist (bicuculline, 10 μM, Figure 1C). EPSCs, but not IPSCs, followed high-frequency (20 Hz) synaptic stimulation reliably (Figure 1D). EPSCs had a fixed latency whereas the latencies of IPSCs showed larger variability (Figure 1E and 1F). Average latency of EPSCs (from stimulus artifact to onset of synaptic current) was significantly shorter than that of IPSCs (see individual example in Figure 1F; data are summarized in Figure 1G, n = 10 neurons; P < 0.01, paired t-test). Failure to follow high-frequency synaptic stimulation was significant in the sample of neurons (n = 15; P < 0.01, Dunnett's multiple comparison test, Figure 1H). The data show that CeLC neurons receive monosynaptic excitatory and polysynaptic inhibitory inputs from the BLA.

**Figure 2:**
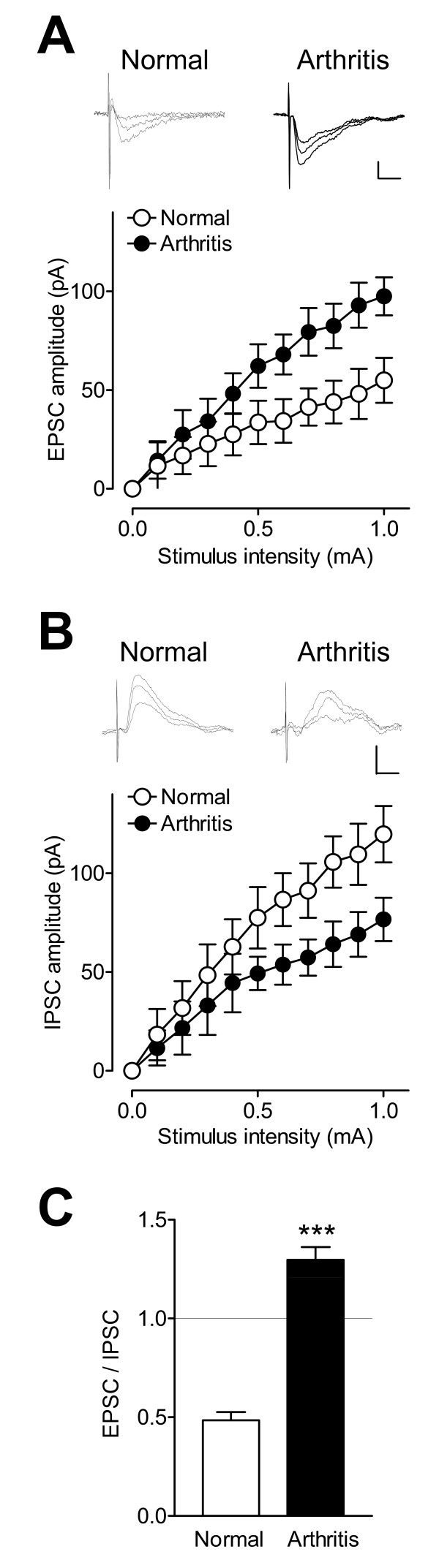
**Increased excitatory and decreased inhibitory transmission in CeLC neurons in a model of arthritic pain**. (**A**) Input-output (I/O) functions of monosynaptic EPSCs (recorded at -70 mV) increased significantly (P < 0.0001, two-way ANOVA, see Results) in slices from arthritis rats (n = 12 neurons) compared with control slices from normal rats (n = 11 neurons). Traces show EPSCs evoked with stimulus intensities of 0.3, 0.6 and 0.9 mA in one CeLC neuron from a normal rat and in another CeLC neuron from an arthritic rat. Scale bars, 50 pA, 10 ms. (**B**) I/O function of IPSCs (recorded at 0 mV) decreased significantly (P < 0.0001, two-way ANOVA, see Results) in slices from arthritic rats (n = 12 neurons) compared with slices from normal rats (n = 11 neurons). Individual traces show IPSCs evoked with stimulation intensities of 0.3, 0.6 and 0.9 mA. Scale bars, 50 pA, 10 ms. (**C**) The ratio of EPSCs and IPSCs evoked with a stimulation intensity of 1 mA increased significantly in slices from arthritic rats (n = 12 neurons) compared to controls (n = 11 neurons). *** P < 0.001, unpaired t-test. (**A-C**) Symbols and error bars represent means ± SE.

To determine pain-related changes of excitatory and inhibitory synaptic inputs, input-output (I/O) relationships were obtained by measuring peak amplitudes of EPSCs and IPSCs as a function of afferent fiber stimulus intensity for each neuron (see examples in Figure 2A and 2B). CeLC neurons were recorded in slices from normal untreated rats and in slices from arthritic rats (4-6 hr after induction of arthritis in one knee joint; see Methods). Compared with controls (n = 11 neurons), I/O function of monosynaptic EPSCs at the BLA-CeLC synapse increased significantly in the arthritis pain model (n = 12 neurons, F_1,231 _= 30.49, P < 0.0001, main effect of treatment, two-way ANOVA; Figure 2A), whereas the I/O function of IPSCs decreased significantly (control, n = 11 neurons; arthritis, n = 12 neurons; F_1,231 _= 22.15, P < 0.0001, main effect of treatment, two-way ANOVA; Figure 2B). The increase of excitatory transmission relative to inhibitory transmission in the arthritis pain model is reflected in the significantly increased EPSC/IPSC ratio (P < 0.001, unpaired t test; Figure 2C).

**Figure 3:**
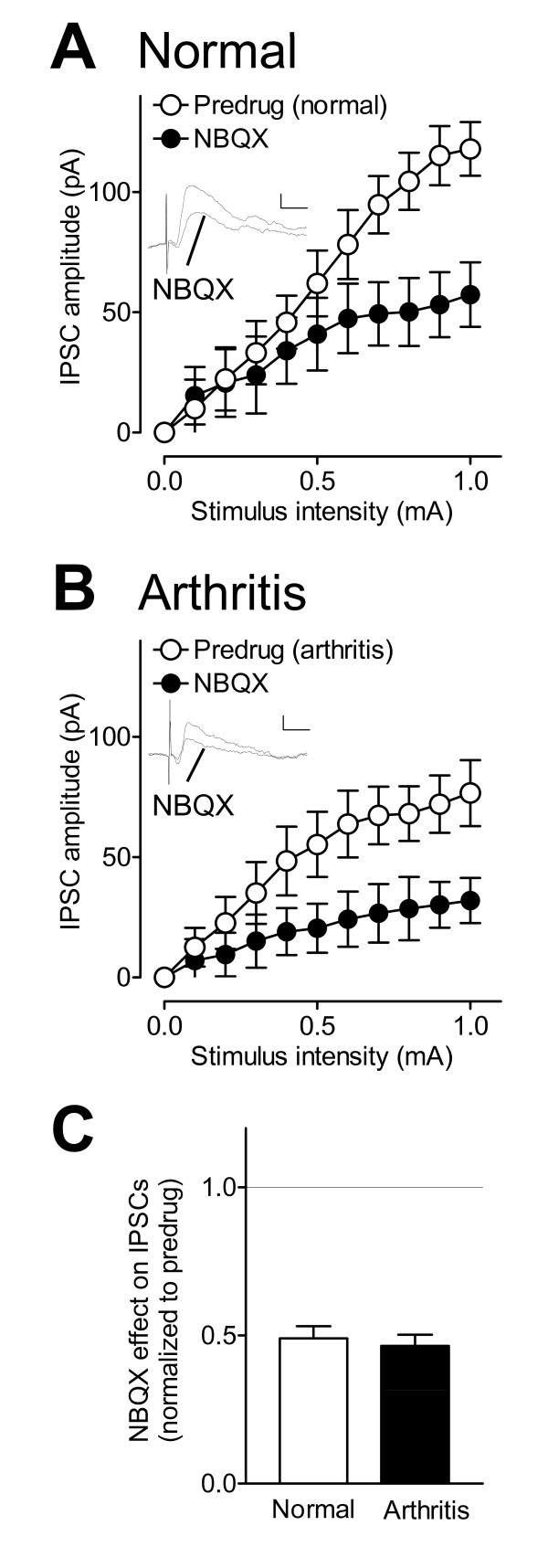
**Inhibitory transmission onto CeLC neurons is driven by non-NMDA receptors**. (**A**) A non-NMDA receptor antagonist (NBQX, 10 μM) decreased input-output (I/O) functions of IPSCs (recorded at 0 mV) in slices from normal rats significantly (n = 5 neurons, P < 0.0001, two-way ANOVA; see Results). Traces show IPSCs evoked in one CeLC neuron before (Predrug) and during NBQX. Stimulus intensity, 0.9 mA; scale bars, 50 pA, 10 ms. (**B**) NBQX also decreased I/O function of IPSCs (recorded at 0 mV) in slices from arthritic rats (n = 5 neurons) significantly (n = 5 neurons, P < 0.0001, two-way ANOVA; see Results). Individual traces show IPSCs evoked in one CeLC neuron before (Predrug) and during NBQX. Stimulus intensity, 0.9 mA; scale bars, 50 pA, 10 ms. (**C**) The effect of NBQX normalized to predrug values (set to 1.0) was not significantly different between slices from arthritic rats and normal controls (P > 0.05, unpaired t-test). (**A-C**) Symbols and error bars represent means ± SE.

Next we tested the hypothesis that polysynaptic inhibitory transmission is glutamate- driven. IPSCs were inhibited by a non-NMDA receptor antagonist (NBQX, 10 μM; Figure 3) in slices from normal animals (n = 5 neurons, F_1,88 _= 24.11, P < 0.0001, main effect of drug, two-way ANOVA; Figure 3A) and in slices from arthritic rats (n = 5 neurons, F_1,88 _= 36.18, P < 0.0001, main effect of drug, two-way ANOVA; Figure 3B). The effect of NBQX on I/O functions of inhibitory transmission was not significantly different in arthritis compared to normal conditions (P > 0.05, unpaired t-test; Figure 3C). The pharmacological profile (blockade by NBQX) and synaptic characteristics (longer and more variable latencies and inability to follow high-frequency stimulation) indicate that IPSCs recorded in CeLC neurons are polysynaptic, involving a glutamatergic synapse. The results are consistent with morphological and functional evidence for glutamatergic projections from the BLA to GABAergic interneurons in the ITC [2,35] and suggest that CeLC neurons receive disynaptic feedforward inhibition.

### Pain-related loss of GABAergic inhibition of excitatory transmission

**Figure 4:**
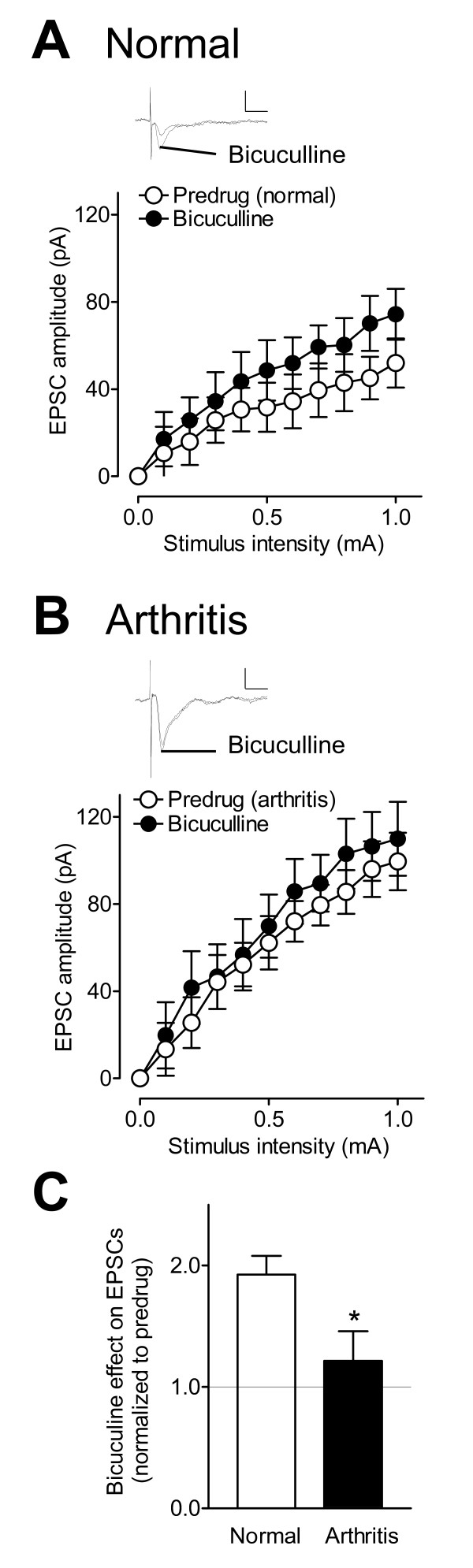
**GABAergic inhibition is lost in a model of arthritic pain**. (**A**) A GABA_A _receptor antagonist (bicuculline, 10 μM) significantly increased I/O function of EPSCs evoked in CeLC neurons in slices from normal rats (n = 5 neurons, P < 0.001, two-way ANOVA; see Results). Individual traces show EPSCs evoked in one CeLC before (Predrug) and during bicuculline. Stimulus intensity, 0.9 mA. Scale bars: 50 pA, 10 ms. (**B**) Bicuculline had no significant effect on EPSCs recorded in CeLC neurons in slices from arthritic rats (n = 5 neurons, P > 0.05, two-way ANOVA; see Results). Traces show EPSCs recorded in one CeLC neuron before (Predrug) and during bicuculline; they were nearly identical as bicuculline had no effect. Stimulus intensity, 0.9 mA; scale bars, 50 pA, 10 ms. (**C**) Bar histograms show the significantly greater facilitatory effect of bicuculline on EPSCs (normalized to predrug; set to 1.0) in slices from normal rats compared to arthritic rats. * P < 0.05, unpaired t- test. (**A-C**) Symbols and error bars represent means ± SE.

We sought to determine if feedforward inhibition of CeLC neurons modulates excitatory synaptic transmission from the BLA and if that effect changes in the arthritis pain state. A GABA_A _receptor antagonist (bicuculline, 10 μM) significantly increased I/O function of excitatory transmission at the BLA-CeLC synapse under normal conditions (n = 5 neurons, F_1,88 _= 8.80, P < 0.01, main effect of drug, two-way ANOVA; Figure 4A), suggesting GABAergic control of excitatory inputs to the CeLC. In slices from arthritic rats, however, bicuculline had no significant effect on EPSCs evoked in CeLC neurons (n = 5 neurons, F_1,88 _= 2.67, P > 0.05, main effect of drug, two-way ANOVA; Figure 4B). The significantly decreased facilitatory effect of bicuculline in the arthritis model (P < 0.05, unpaired t-test; Figure 4C) may suggest that loss of inhibition contributes at least in part to the pain-related increase of excitatory transmission (see Figure 2A).

### mGluR1, but not mGluR5, can account for the pain-related changes of excitatory and inhibitory transmission in CeLC neurons

**Figure 5:**
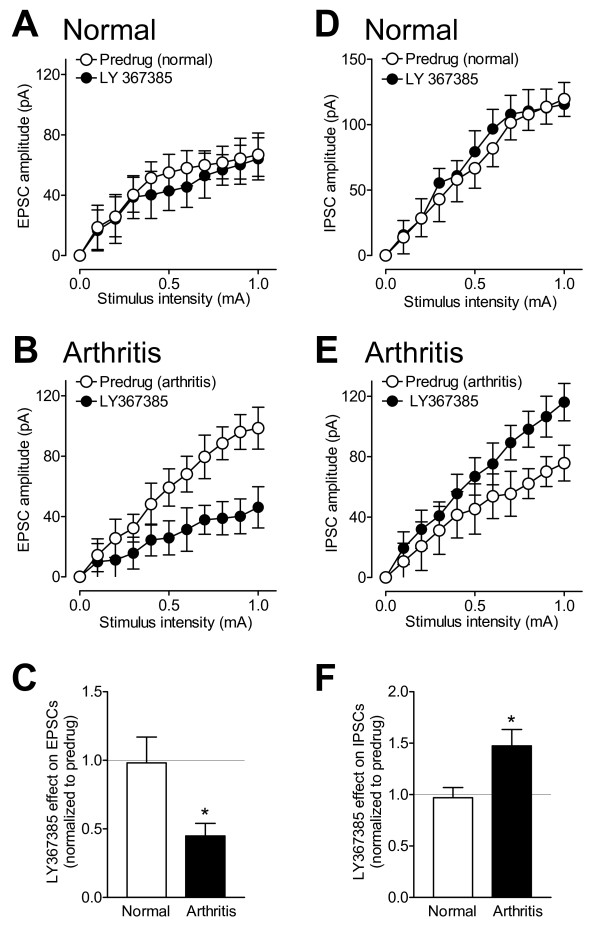
**Blockade of mGluR1 reverses the pain-related increase of excitatory transmission and the decrease of inhibitory transmission in CeLC neurons**. (**A**) A selective mGluR1 antagonist (LY367385, 10 μM) had no significant effect on I/O functions of EPSCs at the BLA-CeLC synapse in slices from normal rats (n = 3 neurons, P > 0.05, two-way ANOVA; see Results). (**B**) LY367385 inhibited excitatory transmission in slices from arthritic rats (n = 5 neurons, P < 0.0001, two-way ANOVA; see Results). (**C**) The inhibitory effect of LY367385 (normalized to predrug; set to 1.0) in the arthritis pain model was significantly different from that under normal conditions. * P < 0.05, unpaired t-test. (**D**) LY367385 had no significant effect on I/O functions of IPSCs in slices from normal rats (n = 3 neurons, P > 0.05, two-way ANOVA; see Results). (**E**) LY367385 increased inhibitory transmission in slices from arthritic rats significantly (n = 5 neurons, P < 0.0001, two-way ANOVA; see Results). (**F**) The facilitatory effect of LY367385 (normalized to predrug; set to 1.0) in the arthritis pain model was significantly different from that under normal conditions. * P < 0.05, unpaired t-test. (**A-F**) Symbols and error bars represent means ± SE.

Our previous studies showed that mGluR1 and mGluR5 modulate excitatory transmission in the CeLC and upregulation of mGluR1 is associated with pain-related synaptic plasticity [20]. Here we examined the modulation of inhibitory transmission by mGluR1 and mGluR5. Confirming the results of our previous study obtained with a different mGluR1 antagonist (CPCCOEt) [20], a selective mGluR1 antagonist (LY367385, 10 μM) inhibited excitatory synaptic transmission in CeLC neurons in slices from arthritic rats (n = 5 neurons, F_1,88 _= 37.10, P < 0.0001, main effect of drug, two-way ANOVA; Figure 5B) but had no significant effect under normal conditions (n = 3 neurons, F_1,44 _= 1.03, P > 0.05, main effect of drug, two-way ANOVA; Figure 5A). The inhibitory effect of LY367385 in the pain model was significantly different from that under normal conditions (P < 0.05, unpaired t-test; Figure 5C). LY367385 increased inhibitory synaptic transmission in slices from arthritic rats significantly (n = 5 neurons, F_1,88 _= 15.91, P < 0.0001, main effect of drug, two-way ANOVA; Figure 5E) but had no significant effect under normal conditions (n = 3 neurons, F_1,44 _= 0.56, P > 0.05, main effect of drug, two-way ANOVA; Figure 5D). The facilitatory effect of LY367385 in the pain state was significantly different from that under normal conditions (P < 0.05, unpaired t-test; Figure 5F). The results show that LY367385 can reverse pain-related changes of excitatory as well as inhibitory transmission in the CeLC.

**Figure 6:**
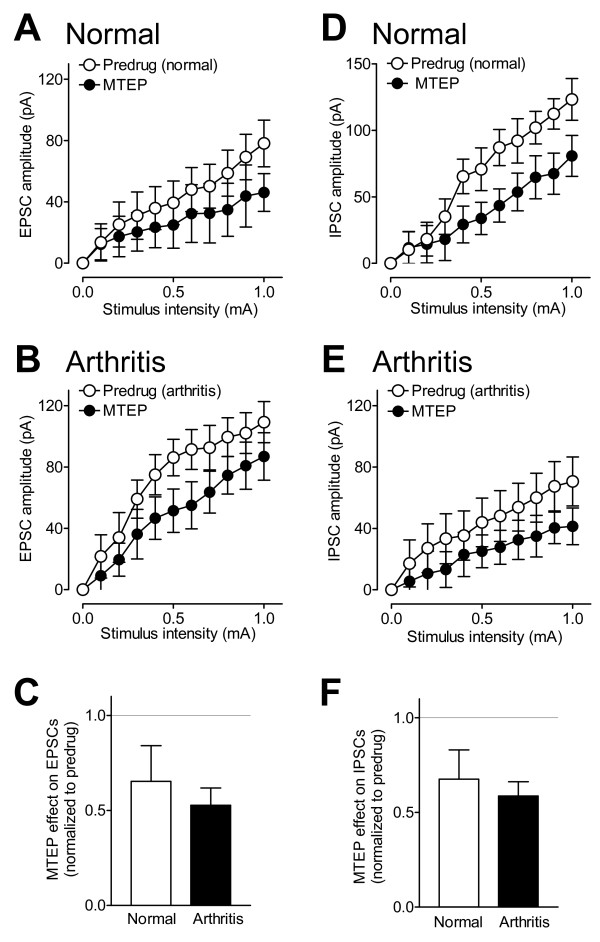
**Blockade of mGluR5 inhibits excitatory and inhibitory transmission under normal conditions and in arthritis**. (**A**) A selective mGluR5 antagonist (MTEP, 1 μM) decreased I/O functions of excitatory transmission in slices from normal rats significantly (n = 5 neurons, P < 0.01, two-way ANOVA; see Results). (**B**) MTEP inhibited EPSCs in slices from arthritic rats significantly (n = 5 neurons, P < 0.0001, two-way ANOVA; see Results). (**C**) Normalized effects (to predrug; set to 1.0) of MTEP on EPSCs were not significantly between arthritis and normal conditions (P > 0.05, unpaired t-test). (**D**) MTEP decreased I/O functions of inhibitory synaptic transmission in slices from normal animals (n = 5 neurons, P < 0.0001, two-way ANOVA; see Results). (**E**) MTEP decreased IPSCs in slices from arthritic rats significantly (n = 5 neurons, P < 0.01, two-way ANOVA; see Results). (**F**) Normalized effects (to predrug; set to 1.0) of MTEP on IPSCs were not significantly different between normal and arthritis conditions (P > 0.05, unpaired t-test). (**A-F**) Symbols and error bars represent means ± SE.

Like the mGluR1 antagonist, a selective mGluR5 antagonist (MTEP, 1 μM) significantly decreased the input-output functions of excitatory synaptic transmission in slices from arthritic rats (n = 5 neurons, F_1,88 _= 16.12, P < 0.0001, main effect of drug, two-way ANOVA; Figure 6B). Unlike LY367385, however, MTEP had inhibitory effects in slices from normal rats (n = 5 neurons, F_1,88 _= 5.75, P < 0.01, main effect of drug, two-way ANOVA; Figure 6A). The inhibitory effects on excitatory transmission were not significantly different between arthritis and normal conditions (P > 0.05, unpaired t-test; Figure 6C). MTEP also decreased inhibitory synaptic transmission in slices from arthritic rats (n = 5 neurons, F_1,88 _= 10.13, P < 0.01, main effect of drug, two-way ANOVA; Figure 6E) and in slices from normal animals (n = 5 neurons, F_1,88 _= 22.54, P < 0.0001, main effect of drug, two-way ANOVA; Figure 6D). The inhibitory effects of MTEP on IPSCs were not significantly different between normal and arthritis conditions (P > 0.05, unpaired t-test; Figure 6F). The results show that mGluR5 are involved in excitatory and inhibitory synaptic transmission under normal conditions and in the pain state, whereas mGluR1 contribute only to the pain-related changes of excitatory and inhibitory transmission in CeLC neurons.

### Presynaptic action potential-dependent action of mGluR1 and postsynaptic action of mGluR5

**Figure 7:**
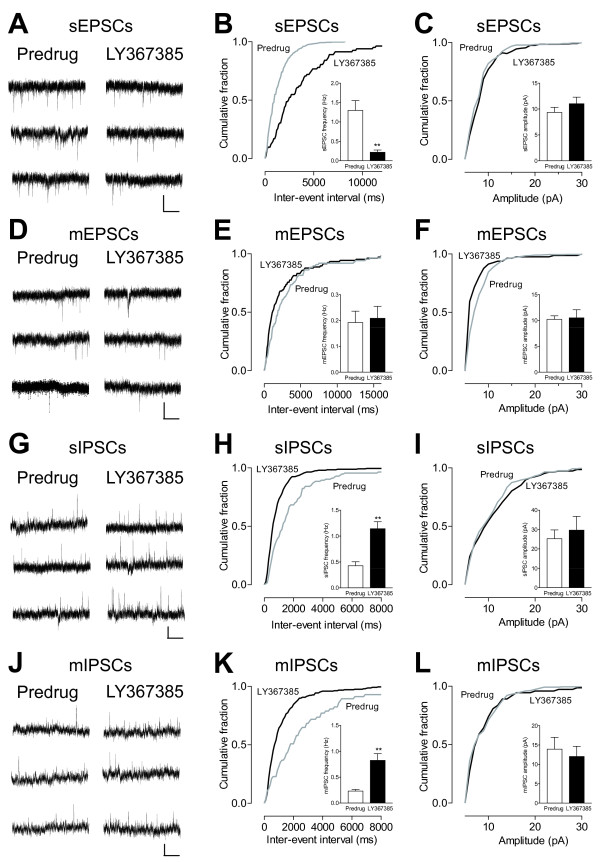
**Effects of LY367385 on spontaneous and miniature excitatory and inhibitory postsynaptic currents in CeLC neurons from arthritic rats**. (**A**) Original current traces of spontaneous EPSCs (sEPSC) in an individual CeLC neuron (held at -70 mV) from an arthritic rat before (Predrug) and during LY367385 (10 μM). (**B, C**) Cumulative distribution analysis of sEPSC frequency (**B**) and amplitude (**C**). LY367385 caused a significant shift toward larger inter-event intervals (lower frequency; P < 0.05, Kolmogorov-Smirnov test). LY367385 decreased mean sEPSC frequency (**B**), but not amplitude (**C**), significantly (n = 6 neurons, P < 0.01, paired t-test). (**D**) Current traces of miniature EPSCs (mEPSCs) recorded in the presence of TTX (1 μM) in one CeLC neuron from an arthritic rat before (Predrug) and during LY367385 (10 μM). (**E, F**) LY367385 (10 μM) had no significant effect on frequency (**E**) and amplitude (**F**) of mEPSC (cumulative distribution, P > 0.05, Kolmogorov-Smirnov test; means ± SE, n = 8 neurons, P > 0.05, paired t-test). (**G**) Current traces of sIPSCs recorded in one CeLC neuron (held at 0 mV) in a brain slice from an arthritic rat before (Predrug) and during LY367385 (10 μM). (**H, I**) LY367385 (10 μM) increased frequency (**H**), but not amplitude (**I**), of sIPSCs significantly (cumulative frequency distribution, P < 0.05, Kolmogorov-Smirnov test; mean frequency, n = 7 neurons, P < 0.01, paired t-test). (**J**) Current traces of mIPSCs in the presence of TTX (1 μM) in one CeLC neuron from an arthritic rat before (Predrug) and during LY367385 (10 μM). (**K, L**) LY367385 (10 μM) increased frequency (**K**), but not amplitude (**L**), of mIPSCs significantly (cumulative frequency distribution, P < 0.05, Kolmogorov-Smirnov test; mean frequency, n = 7 neurons, P < 0.01, paired t-test). (**A, D, G, J**) Scale bars, 10 pA, 2 s. (**B, C, E, F, H, I, K, L**) Bar histograms show means ± SE.

The analysis of spontaneous and miniature EPSCs and IPSCs is a well established electrophysiological approach to determine pre- versus postsynaptic mechanisms. Presynaptic changes at the transmitter release site affect frequency, whereas changes at the postsynaptic membrane would alter amplitude (quantal size) [44]. In slices from arthritic rats, LY367385 (10 μM) decreased frequency (Figure 7B), but not amplitude (Figure 7C), of spontaneous EPSCs (sEPSCs) significantly (cumulative frequency distribution, P < 0.05, Kolmogorov Smirnov test; mean frequency, P < 0.01, paired t-test; n = 6 neurons). Original recordings of sEPSCs are shown in Figure 7A. Recordings were made in slices from arthritic rats, because LY367385 had no significant effect under normal conditions (see Figure 5). LY367385 (10 μM) had no significant effect on frequency (Figure 7E) and amplitude (Figure 7F) of miniature EPSCs (mEPSCs) recorded in TTX (1 μM) in slices from arthritic rats (cumulative distribution, P > 0.05, Kolmogorov-Smirnov test; mean frequency and amplitude, n = 8 neurons, P > 0.05, paired t-test; original traces are shown in Figure 7D). The lack of effect on mEPSCs suggests an action potential-dependent site of action. LY367385 (10 μM) increased frequency, but not amplitude, of sIPSCs (n = 7 neurons; Figure 7G-I) and mIPSCs (n = 7 neurons; Figure 7J-L) significantly (cumulative frequency distribution, P < 0.05, Kolmogorov-Smirnov test; mean frequency, P < 0.01, paired t-test). The data suggest that mGluR1 act presynaptically on GABAergic terminals to regulate glutamatergic transmission in the arthritis pain model.

**Figure 8:**
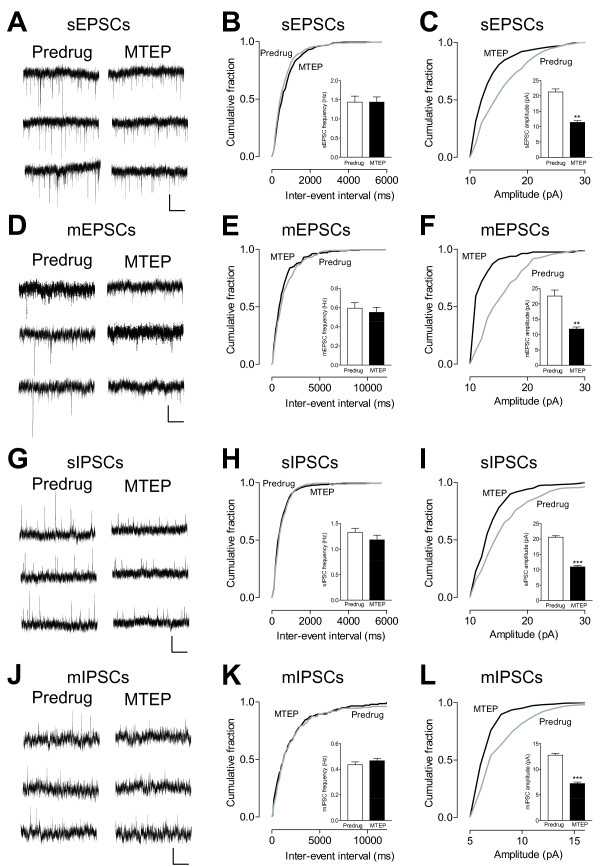
**Effects of MTEP on spontaneous and miniature excitatory and inhibitory postsynaptic currents in CeLC neurons from arthritic rats**. (**A**) Original current traces of sEPSCs in an individual CeLC neuron (held at -70 mV) from an arthritic rat before (Predrug) and during MTEP (1 μM). (**B, C**) MTEP (1 μM) decreased sEPSC amplitude (**C**), but not frequency (**B**), significantly (cumulative amplitude distribution, P < 0.05, Kolmogorov-Smirnov test; mean amplitude, n = 5 neurons, P < 0.01, paired t-test). (**D**) Current traces of mEPSCs recorded in the presence of TTX (1 μM) in one CeLC neuron from an arthritic rat before (Predrug) and during MTEP (1 μM). (**E, F**) MTEP (1 μM) decreased mEPSC amplitude (**F**), but not frequency (**E**), significantly (cumulative amplitude distribution, P < 0.05, Kolmogorov-Smirnov test; mean amplitude, n = 5 neurons, P < 0.01, paired t-test). (**G**) Current traces of sIPSCs recorded in one CeLC neuron (held at 0 mV) in a brain slice from an arthritic rat before (Predrug) and during MTEP (1 μM). (**H, I**) MTEP (1 μM) decreased amplitude (**I**), but not frequency (**H**), of sIPSCs significantly (cumulative amplitude distribution, P < 0.05, Kolmogorov-Smirnov test; mean amplitude, n = 4 neurons, P < 0.001, paired t-test). (**J**) Current traces of mIPSCs in the presence of TTX (1 μM) in one CeLC neuron from an arthritic rat before (Predrug) and during MTEP (1 μM). (**K, L**) MTEP (1 μM) decreased amplitude (**L**), but not frequency (**K**), of mIPSCs significantly (cumulative amplitude distribution, P < 0.05, Kolmogorov-Smirnov test; mean amplitude, n = 4 neurons, P < 0.001, paired t-test). (**A, D, G, J**) Scale bars, 10 pA, 2 s. (**B, C, E, F, H, I, K, L**) Bar histograms show means ± SE.

MTEP (10 μM) decreased the amplitude, but not frequency, of sEPSCs (n = 5 neurons; Figure 8A-C) and mEPSCs (n = 5 neurons; Figure 8D-F) recorded in CeLC neurons in slices from arthritic rats (cumulative amplitude distribution, P < 0.05, Kolmogorov-Smirnov test; mean amplitude, P < 0.01, paired t-test). MTEP also decreased the amplitude (n = 4 neurons; Figure 8G-I), but not frequency (n = 4 neurons; Figure 8J-L), of sIPSCs and mIPSCs in CeLC neurons significantly (cumulative amplitude distribution, P < 0.05, Kolmogorov-Smirnov test; mean amplitude, P < 0.001, paired t-test). The data suggest that mGluR5 regulate both excitatory and inhibitory synaptic transmission in CeLC neurons through a postsynaptic mechanism of action.

### Presynaptic modulation of excitatory transmission by mGluR1 involves GABAA receptors

**Figure 9:**
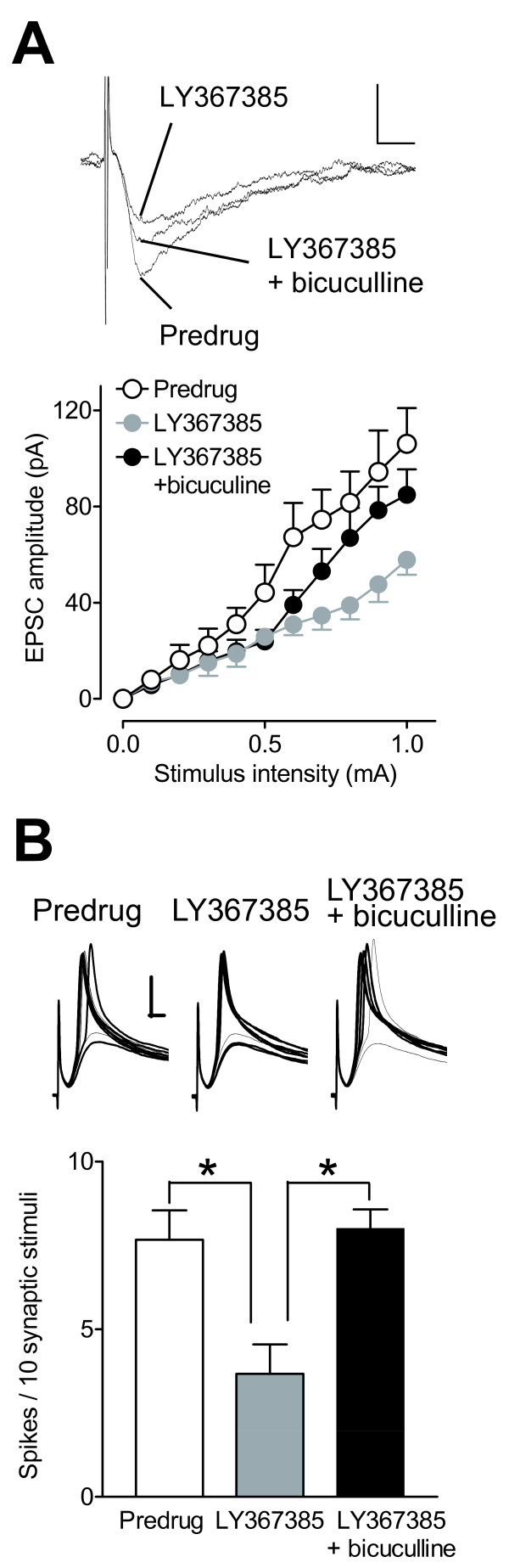
**Blockade of mGluR1 restores facilitatory effects of bicuculline on excitatory transmission in the arthritis pain model**. (**A**) LY367385 (10 μM) decreased I/O function of excitatory synaptic transmission significantly (n = 5 neurons, P < 0.0001, main effect of drug, two-way ANOVA; see Results). Coapplication of bicuculline (10 μM) partially reversed the inhibitory effect of LY367385 (n = 5 neurons, P < 0.001, main effect of drug, two-way ANOVA; see Results). Current traces of EPSCs recorded in one CeLC neuron in a brain slice from an arthritic rat before (Predrug) and during application of LY367385 alone and together with bicuculline. Scale bars, 50 pA, 10 ms. (**B**) Current-clamp recordings show that LY367385 (10 μM) decreased the number of synaptically evoked action potentials in CeLC neurons in slices from arthritic rats (n = 5 neurons, P < 0.05, ANOVA with Bonferroni posttest). The addition of bicuculline (10 μM) partially reversed the inhibitory effect of LY367385 (n = 5 neurons, P < 0.05, ANOVA with Bonferroni posttest). Bar histograms show the number of spikes per 10 synaptic stimuli at near-threshold stimulus intensity averaged across the sample of neurons. Original traces show action potentials and excitatory postsynaptic potentials evoked in an individual CeLC neuron in a slice from an arthritic rat. Scale bars, 20 mV, 5 ms. (**A, B**) Symbols and error bars represent means ± SE.

The data presented so far show that mGluR1 acts presynaptically to inhibit GABAergic transmission but also increases excitatory transmission through an action potential dependent "presynaptic" mechanism. We tested the hypothesis that the inhibitory action of mGluR1 on GABAergic terminals is a mechanism by which mGluR1 increase excitatory transmission. LY367385 (10 μM) decreased excitatory synaptic transmission (EPSCs, n = 5 neurons, F_1,88 _= 42.06, P < 0.0001, main effect of drug, two-way ANOVA; Figure 9A) and the number of synaptically evoked spikes (n = 5 neurons, P < 0.05, ANOVA with Bonferroni posttest; Figure 9B) in CeLC neurons in slices from arthritic rats. The addition of bicuculline (10 μM) partially reversed the inhibitory effect of LY367385 on EPSCs (n = 5 neurons, F_1,88 _= 14.85, P < 0.001, main effect of drug, two-way ANOVA; Figure 9A) and on synaptically evoked spikes (n = 5 neurons, P < 0.05, ANOVA with Bonferroni posttest; Figure 9B), whereas bicuculline alone had no effect in the arthritis pain state (see Figure 4B). The data suggest that removal of the mGluR1-mediated blockade of inhibitory transmission with an mGluR1 antagonist restores inhibitory control of excitatory synaptic transmission as is evident from the facilitatory effect of a GABAA-receptor antagonist that was lost in the arthritis pain model. Therefore, activation of mGluR1 in arthritis may explain the loss of inhibitory control (disinhibition) of excitatory transmission in the CeLC.

### Monosynaptic IPSCs are not under control of mGluR1 in the arthritis pain model

**Figure 10:**
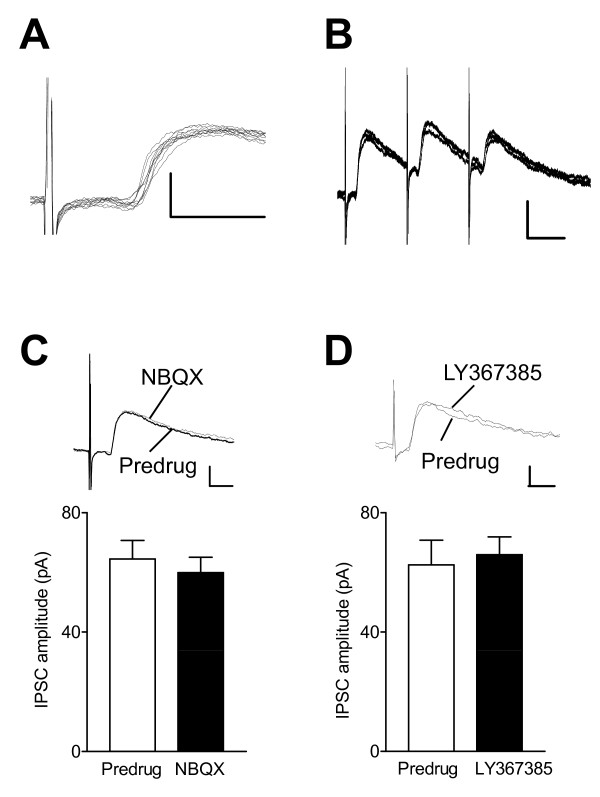
**Blockade of mGluR1 has no effect on monosynaptic IPSCs in the arthritis pain model**. (**A, B**) Individual traces of monosynaptic IPSCs recorded in one CeLC neuron at 0 mV show little variability in latency (10 sweeps) and follow high-frequency stimulation (20 Hz, 6 sweeps). Scale bars 50 pA, 10 ms. (**C**) NBQX (10 μM) had no significant effect on monosynaptic IPSCs (n = 3 neurons; P > 0.05, paired t-test). (**D**) LY367385 (10 μM) had no significant effect on monosynaptic IPSCs (n = 3 neurons; P > 0.05, paired t-test). (**C, D**) Individual traces show monosynaptic IPSCs before (Predrug) and during drug application. Stimulus intensity, 0.9 mA; scale bars, 50 pA, 10 ms. Bar histograms show means ± SE.

IPSCs evoked in some CeLC neurons showed little variability in latency and followed high-frequency stimulation, suggesting that they were monosynaptic (Figure 10A and 10B). NBQX (10 μM) had no significant effect on monosynaptic IPSCs (n = 3 neurons, P > 0.05, paired t-test; Figure 10C). In contrast to its facilitatory effect on polysynaptic IPSCs associated with feedforward inhibition of CeLC neurons (Figure 5E and 5F), LY367385 (10 μM) had no significant effect on monosynaptic IPSCs in slices from arthritic rats (n = 3 neurons, P > 0.05, paired t-test; Figure 10D). The origin of these monosynaptic inhibitory inputs remains to be determined.

## Discussion

**Figure 11:**
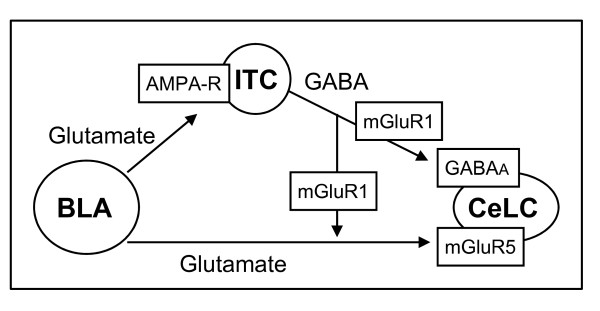
**Proposed circuitry**. CeLC neurons receive direct excitatory input from the lateral-basolateral (BLA) nuclei that contains highly processed multimodal, including nociceptive, information [5,8]. ITC cells provide inhibitory inputs that contact CeLC directly, acting on GABAA receptors, but also regulate glutamatergic inputs to the CeLC. Presynaptic mGluR1 inhibit GABAergic afferents. In the pain state, increased glutamatergic transmission to the CeLC activates not only postsynaptic mGluR5 but also presynaptic mGluR1 that inhibit GABAergic transmission. BLA, basolateral amygdala; ITC, intercalated cell masses; CeLC, latero-capsular division of the central nucleus of the amygdala; GABA

The novel key findings of this study on amygdala function related to pain are as follows. 1) In contrast to the increase in excitatory synaptic transmission, inhibitory feedforward inhibition of CeLC neurons decreases in a model of arthritis pain, shifting the balance toward a dominance of excitatory inputs. 2) The differential change of excitatory and inhibitory transmission involves mGluR1 acting presynaptically on GABAergic terminals to decrease inhibitory and enhance excitatory transmission. 3) Postsynaptic mGluR5 contribute to both excitatory and inhibitory synaptic transmission under normal conditions and in the arthritis pain model; their effect on synaptic inputs does not change in the pain state. To conclude, the data confirm the results of our previous studies [20,27] that a change in the function of mGluR1 rather than mGluR5 contributes to enhanced excitatory transmission and increased activity of CeLC neurons; importantly, the present study provides novel insight into underlying mechanism by showing that mGluR1 exert their facilitatory effect through disinhibition, i.e., inhibition of inhibitory control of excitatory inputs to the CeLC (see Figure 11).

The conclusion is supported by the following observations. We show for the first time a change in feedforward inhibition of CeLC neurons in a pain model. Pharmacological evidence (blockade by NBQX) and synaptic characteristics (longer and more variable latencies and inability to follow high-frequency stimulation reliably) indicate that synaptic inhibition of CeLC neurons is polysynaptic, involving a glutamatergic synapse. The results are consistent with reports in the literature that glutamatergic projections from the BLA do not only reach the CeA directly but also target a cluster of GABAergic interneurons in the ITC that are interposed between BLA and CeA [2,18,35,45,46]. The CeA serves as the output nucleus for major amygdala functions and regulates behavioral responses through projections to hypothalamic nuclei and brainstem areas [4,45,47]. The CeA receives affect-related information that is generated in the LA-BLA network through associative processes [3,4,18,32]. BLA axons projecting toward the CeA form excitatory synapses with ITC neurons that project to the CeA where they generate feed-forward inhibition [45,48,49]. Accumulating evidence suggests that direct glutamatergic projections from the BLA to the CeA are important for fear expression whereas activation of ITC neurons that inhibit CeA output neurons might account for the reduction of fear expression after extinction [2,35,50,51].

Our results show that feedforward inhibition controls excitatory inputs to the CeA under normal conditions but not in a pain state. The fact that bicuculline enhanced excitatory transmission while the CeLC neuron was held at -70 mV, the equilibrium potential for chloride ions, may suggest that GABAA-receptors modulate excitatory inputs indirectly through a "presynaptic" site of action or in the network rather than on the CeLC neuron itself. The significantly decreased facilitatory effect of bicuculline in the arthritis model suggests that a loss of inhibition that may contribute at least in part to the pain-related increase of excitatory transmission mediated by mGluR1.

The results obtained with highly selective antagonists for mGluR1 and mGluR5 [40,42,52,53] show that presynaptic mGluR1, but not postsynaptic mGluR5, can account for the pain-related changes of excitatory and inhibitory transmission in CeLC neurons. The interaction between mGluR1 and GABAergic transmission further suggests that pain-related decrease of inhibitory transmission is an active process that involves activation of mGluR1. Based on the analysis of spontaneous and miniature synaptic events, mGluR1 act presynaptically on GABAergic inputs whereas their effect on excitatory transmission is indirect through a process that requires action potential dependent network activity. Excitatory transmission is under GABAergic inhibition that is lost in arthritis through a mechanism that involves mGluR1. Removal of the mGluR1-mediated blockade of inhibitory transmission with an mGluR1 antagonist restores inhibitory control of excitatory synaptic transmission. Therefore, activation of mGluR1 that is seen in arthritis but not under normal conditions may explain the loss of inhibitory control (disinhibition) of excitatory transmission in the CeLC.

Group I mGluR subtypes mGluR1 and mGluR5 play important roles in physiological neuroplasticity as well as in neurological and psychiatric disorders [36-39] and in pain mechanisms [40-42]. Our previous studies showed that in the amygdala, activation of mGluR5, but not mGluR1, enhanced the excitatory responses of CeLC neurons to innocuous and noxious stimuli in naïve animals [54,55]. In the arthritis pain model, blockade of mGluR1 and mGluR5 decreased the enhanced activity of CeLC neurons to normal-like levels, suggesting a major change in the function of mGluR1 in pain. The underlying mechanism included presynaptic facilitation of excitatory transmission from the parabrachial area and the BLA to CeLC neurons by mGluR5 under normal conditions and by mGluR1 and mGluR5 in the arthritis pain model [20]. Largely based on agonist data we assumed that both mGluR1 and mGluR5 acted presynaptically in that model. However, the detailed analysis of miniature events in the present study suggests that mGluR5 are postsynaptic and mGluR1 have presynaptic effects. The new results further show that the action of mGluR1 on excitatory transmission involves the inhibition of disynaptic inhibitory inputs from the BLA (disinhibition). Inhibition of inhibitory synaptic transmission by group I mGluRs has been shown in the hippocampus (mGluR1 [56,57]), striatum (mGluR1 [58]), cerebellum (mGluR1 [59]), midbrain [60,61] and periaqueductal gray (mGluR5 [62]). The mechanism of inhibition was typically presynaptic, and some evidence suggests the involvement of retrograde endogenous cannabinoid signaling through CB1 receptors [62-64].

## Conclusion

Both increased excitatory transmission and decreased inhibitory transmission in the CeLC in a model of arthritis pain involve mGluR1. These receptors act presynaptically to decrease synaptic inhibition, thus dis-inhibiting excitatory inputs to the CeLC, which may explain the loss of inhibitory control and increase in excitatory transmission observed in the arthritis pain model. mGluR5 act postsynaptically to facilitate both excitatory and inhibitory inputs, but they cannot account for the differential pain-related changes that involve loss of presynaptic GABAergic control of excitatory transmission to the CeLC. The concept of disinhibition of amygdala function may provide important insights into emotional-affective pain mechanisms and potential therapeutic strategies.

## Methods

### Animals

Male Sprague Dawley rats (120-250 g) were individually housed in standard plastic cages (40 × 20 cm) in a temperature-controlled room and maintained on a 12 hr day/night cycle. Standard laboratory chow and tap water were available ad libitum. On the day of the experiment, rats were transferred from the animal facility and allowed to acclimate to the laboratory for at least 1 hr.

### Arthritis pain model

In one group of rats ("arthritis"), arthritis was induced in one knee joint as described in detail previously [65]. A kaolin suspension (4%, 80-100 μl) was slowly injected into the joint cavity through the patellar ligament with a syringe and needle (1 ml,). After repetitive flexions and extensions of the knee for 15 min, a carrageenan solution (2%, 80-100 μl) was injected into the knee joint cavity, and the leg was flexed and extended for another 5 min. This treatment paradigm reliably leads to localized inflammation and swelling of the injected knee within 1-3 hr. The inflammation persists for up to 2 weeks. It does not spread systemically [65]. Another group of rats ("normal") did not receive any injections but was kept under the same conditions as the arthritis rats before brain slices were obtained for electrophysiological studies.

### Electrophysiology

#### Amygdala slice preparation

Brain slices containing the CeA were obtained from normal rats and from arthritic rats (4-6 h after arthritis induction) as described before [10,20,21,23]. Rats were decapitated, and the brains quickly were dissected out and blocked in cold (4°C) artificial cerebrospinal fluid (ACSF). ACSF contained the following (in mM): 117 NaCl, 4.7 KCl, 1.2 NaH_2_PO_4_, 2.5 CaCl_2_, 1.2 MgCl_2_, 25 NaHCO_3_, and 11 glucose. ACSF was oxygenated and equilibrated to pH 7.4 with a mixture of 95% O_2_/5% CO2. Coronal brain slices (500 μm) were prepared using a Vibroslice (Camden Instruments, London, UK). After incubation in ACSF at room temperature (21°C) for at least 1 h, a single brain slice was transferred to the recording chamber and submerged in ACSF (31 ± 1°C), which superfused the slice at 2 ml/min.

#### Whole-cell patch-clamp recording

Whole-cell voltage-clamp recordings were made from CeLC neurons (see Figure 1A and 1B) in brain slices from normal and arthritic rats using the "blind" patch technique as in our previous studies [10,20,21,23]. One neuron was recorded in each slice and 1 or 2 slices were used per animal. Patch electrodes (4-6 MΩ tip resistance) were made from borosilicate glass capillaries (1.5 and 1.12 mm, outer and inner diameter, respectively; Drummond, Broomall, PA) pulled on a Flaming-Brown micropipette puller (P-97/PC; Sutter Instruments, Novato, CA). The internal solution of the recording electrodes contained (in mM): 122 K-gluconate, 5 NaCl, 0.3 CaCl_2_, 2 MgCl_2_, 1 EGTA, 10 HEPES, 5 Na_2_-ATP, and 0.4 Na_3_-GTP, pH was adjusted to 7.2-7.3 with KOH and osmolarity to 280 mOsm/kg with sucrose. Data acquisition of current signals was done using a dual four-pole Bessel filter (Warner Instruments), a low-noise Digidata 1322 interface (Molecular Devices), an Axoclamp-2B amplifier (Molecular Devices) and a Pentium personal computer. Evoked current data were acquired and analyzed using pCLAMP10 software (Axon Instruments). Head-stage voltage was monitored continuously on an oscilloscope to ensure precise performance of the amplifier. Neurons were voltage-clamped at -70 (chloride reversal potential) or 0 mV (reversal potential of EPSCs) for the study of excitatory and inhibitory transmission, respectively. High gigaohm seal and low series (20 MΩ) resistances were checked throughout the experiment (using pClamp9 membrane test function) to ensure high-quality recordings.

#### Synaptic transmission

Excitatory and inhibitory postsynaptic currents (EPSCs and IPSCs, respectively) were evoked in CeLC neurons (held at -70 mV or 0 mV) by electrical stimulation (150 μs square-wave pulses; S88 stimulator; Grass Instruments) of BLA afferents (see Figure 1B) using a concentric bipolar stimulating electrode (David Kopf Instruments). The distance between stimulation and recording electrode was about 1 mm. Input-output relationships were obtained by increasing the stimulus intensity in 0.1 mA steps. For evaluation of a drug effect on synaptically evoked responses, the stimulus intensity was adjusted to 80% of the intensity required for the maximum response. Spontaneous and miniature (in 1 μM TTX) EPSCs and IPSCs were recorded at -70 and 0 mV, respectively [23]. A fixed length of traces (5 min) was analyzed for frequency and amplitude distributions using MiniAnalysis program 5.3 (Synaptosoft). The root mean square (RMS) of the background noise was computed for each set of data. The detection threshold for an event was set to 3-4 times the RMS value. Peaks were detected automatically, but each detected event was then visually inspected to avoid the inclusion of false data.

### Drugs

The following drugs were used: 2,3-dioxo-6-nitro-1,2,3,4-tetrahydrobenzo[f]quinoxaline-7- sulfonamide disodium salt (NBQX; non-NMDA receptor antagonist); bicuculline(GABAA receptor antagonist); α-amino-4-carboxy-2-methylbenzeneacetic acid (LY367385; selective mGluR1 antagonist) and 3-((2-Methyl-1,3-thiazol-4-yl)ethynyl)pyridine hydrochloride (MTEP; selective mGluR5 antagonist). Drugs were purchased from Tocris Cookson (Bristol, UK). All drugs were dissolved in ACSF to their final concentration on the day of the experiment. Selectivity and target concentrations have been established in the literature [40,42,52,53]. Drugs were applied to the brain slice by gravity-driven superfusion in the ACSF. ACSF contained (in mM): 117 NaCl, 4.7 KCl, 1.2 NaH_2_PO4, 2.5 CaCl_2_, 1.2 MgCl_2_, 25 NaHCO_3_, and 11 glucose. Solution flow into the recording chamber (1 ml volume) was controlled with a three-way stopcock. Drugs were applied for at least 8-10 min to establish equilibrium in the tissue. ACSF served as vehicle control in all experiments.

### Statistical analysis

All averaged values are given as the mean ± SE. Statistical significance was accepted at the level P < 0.05. GraphPad Prism 3.0 software (Graph-Pad Software, San Diego, CA) was used for all statistical analysis. For multiple comparisons of I/O functions, one-way ANOVA or two-way ANOVA was used with appropriate posttests (Bonferroni to compare selected pairs of data; Dunnett's multiple comparison test to compare all data to a control value). Student's t test (paired or unpaired when appropriate) was used to compare two sets of data that have Gaussian distribution and similar variances. Kolmogorov-Smirnov test was used for cumulative distribution analysis of spontaneous and miniature synaptic events (MiniAnalysis program 5.3, Synaptosoft Inc., Decatur, GA).

## List of abbreviations

BLA: basolateral nucleus of the amygdale; CeA: central nucleus of the amygdale; CeLC: latero-capsular division of the CeA; EPSC: excitatory postsynaptic current; IPSC: inhibitory postsynaptic current; ITC: intercalated cell masses; LA: lateral nucleus of the amygdale; mEPSC: miniature EPSC; mIPSC: miniature IPSC; sEPSC: spontaneous EPSC; sIPSC: spontaneous IPSC

## Competing interests

The authors declare that they have no competing interests.

## Authors' contributions

WR performed patch-clamp recordings, analyzed electrophysiology data, provided figures and wrote the first draft of the manuscript. VN conceptualized the hypothesis, designed and supervised the experiments, directed the data analysis, and finalized the manuscript. All authors read and approved the manuscript.
